# The skimmed milk proteome of dairy cows is affected by the stage of lactation and by supplementation with polyunsaturated fatty acids

**DOI:** 10.1038/s41598-024-74978-1

**Published:** 2024-10-14

**Authors:** Arash Veshkini, Harald M. Hammon, Laura Vogel, Didier Viala, Mylène Delosière, Arnulf Tröscher, Sébastien Déjean, Fabrizio Ceciliani, Helga Sauerwein, Muriel Bonnet

**Affiliations:** 1 Institute of Animal Science, Physiology Unit, University of Bonn, Bonn, Germany; 2 Research Institute for Farm Animal Biology (FBN), 18196 Dummerstorf, Germany; 3 INRAE, Université Clermont Auvergne, VetAgro Sup, UMR Herbivores, 63122 Saint-Genès-Champanelle, France; 4 Department of Veterinary Medicine, Università degli Studi di Milano, Lodi, Italy; 5 BASF SE, 68623 Lampertheim, Germany; 6 Institut de Mathématiques de Toulouse, UMR 5219, Université de Toulouse, CNRS, UPS, 31062 Toulouse, France

**Keywords:** Biological techniques, Computational biology and bioinformatics, Molecular biology, Physiology, Zoology

## Abstract

The impact of nutritional modification to increase functional polyunsaturated fatty acids (PUFA), such as n-3 and n-6 fatty acids (FA) or conjugated linoleic acid (CLA), on milk proteome profile during early lactation remains largely unknown. We used an untargeted proteomics approach to investigate the impact of lactation day and PUFA supplementation on the proteome signature in skimmed milk over the course of early lactation. Sixteen Holstein dairy cows received abomasal infusion of saturated FA (CTRL) or a mixture of essential FA and CLA (EFA + CLA group) from − 63 to + 63 days relative to parturition. Using quantitative proteomics, 479 unique proteins were identified in skimmed milk at days 1, 28, and 63 postpartum. The top discriminating proteins between transition milk (day 1) and mature milk (days 28 and 63), including members of complements (i.e. C2 and C5), growth factor (TGFB2), lipoproteins (i.e. APOE and APOD), and chaperones (i.e. ST13 and CLU), are associated with calves’ immune system and gut development. The EFA + CLA supplementation moderately affected a few proteins associated with regulating mammary glands’ lipogenesis through the (re)assembly of lipoprotein particles, possibly under the PPAR signaling pathway. Collectively, skimmed milk proteome is dynamically regulated initially by cow’s metabolic and physiological changes and to a lesser extent by nutritional PUFA modifications.

**Supplementary Information:**

The online version contains supplementary material available at 10.1038/s41598-024-74978-1.

## Introduction

Bovine colostrum (1st mammary secretion after calving), transition milk (2nd to 10th milking), and mature milk (> 10th milking) contain nutrients such as carbohydrates, lipids, and proteins as well as diverse bioactive compounds including vitamins, growth factors, hormones, and oligosaccharides, proportional to health and developmental requirements of the calves^1,2^. In cattle as in most ungulates, the newborn offspring depends on the passive immunization via colostrum since the epitheliochorial placenta type in these species prevents the transfer of immunoglobulins (Ig) from the maternal to the fetal circulation^3^. The Ig can make up 70 − 80% of the total protein content of bovine colostrum, whereas mature milk contains only 1–2%^4^. In addition to lactation day, colostrum and milk composition are influenced by a number of factors, including diet, supplements, and animal health status, which can further affect calf growth and development^5,6^.

Advanced liquid chromatography and mass spectrometry (LC-MS/MS) technologies provide robust and reliable tools for investigating the proteome fingerprint of mammary secretions and their gradual transition from colostrum to mature milk. In this regard, a previous paper identified exclusive milk and colostrum proteins and sorted them based on their possible impact on calves’ health^7^. The author concluded that there are many other important proteins involved in calves’ growth and immunity besides Ig, including complement factors (e.g., C4, C6, and C9), antimicrobial proteins (e.g., collectin-43 and conglutinin), acute phase proteins (e.g., haptoglobin and milk amyloid A) and apolipoprotein classes (APOE, APOA1). Later on, the proteomes of colostrum and transition milk from primiparous and multiparous cows were characterized and grouped based on their ontology^8^. The proteome profiles of colostrum and mature milk from the first and second lactation were recently investigated^9^. Furthermore, proteomics analyses suggested specific milk proteins as putative indicators of postpartum negative energy balance (NEB) and metabolic imbalance in dairy cows^10^.

Nutritional modifications to increase beneficial fatty acids (FA) such as essential fatty acids (EFA) and conjugated linoleic acids (CLA) have been gathering extra attention as a strategy to naturally improve the energy status of dairy cows, as well as their metabolic health during transitioning to lactation^11,12^. Namely, EFA including linoleic acid (LA, C18:2 n-6) and α-linolenic acid (ALA, C18:3, n-3), are involved in key biological systems such as immune function, inflammation, oxidative stress, signal transduction, and gene expression regulation. On the other hand, CLA, the naturally occurring conjugated form of LA, induce milk fat depression on top of that^13^. As a result, CLA-treated cows reached a positive EB ~ 3 weeks earlier than those receiving saturated fats^14^. In addition, CLA supplementation reduced the size of milk fat globules (MFG)^15^, which consist of a triglyceride (TG) core surrounded by phospholipids and proteins that stabilise milk FAs^16^. CLA supplementation also altered the MFG membrane protein profile, which is involved in the determination of MFG size^17^. It has been shown that FA supplementation affects cow milk composition, such as fat, protein, urea, and FA^18,19^. While few experimental studies have reported reduced milk protein percentages, milk protein content is generally among the most stable components and is less affected by nutritional FA modifications^12^. For instance, in cows fed increasing amounts of extruded flaxseed (from 5 to 15% of dry matter)^20^ or whole flaxseed^21^ as a rich source of n-3 FA, milk protein percentage was not affected. It has also been found that supplementation of low, medium, and high levels of rumen-protected CLA (50, 60, 100, 150, 200, 400, and 600 g/d) during the transition period did not affect milk protein levels significantly^22–25^. This might be due to the predominant level of caseins, which represent about 80% of total bovine milk protein content^26^. However, little is known about the low-abundance proteome profile of milk due to the recent availability of proteomic technology and recent scientific investigations in this area. According to a recent study, dietary FA supplementation of dairy cows to either increase (corn oil) or decrease (hydrogenated palm oil) milk fat content has impacted milk fat globule membrane (MFG) proteins, which are primarily related to lipid biosynthesis and secretion such as fatty acid binding protein (FABP3) and solute carrier family members (SLC34A2 and SLC39A8)^27^.

The skimmed milk fraction, after MFG removal, contains an array of active proteins^28,29^ including FA sensing elements and transporters, which are involved in metabolism, transportation, esterification, and storage of FA in the cells and in body fluids; the proteins’ transcriptional regulation is also affected by FA^30^. Investigating milk improvement strategies, such as EFA and CLA enrichment, on the proteome signature of mammary secretions during early lactation might open up new opportunities to achieve and direct the calves’ developmental competence. This study investigated the proteome profile in skimmed milk during early lactation and its interaction with FA (EFA and CLA) supplementation. In addition, since early lactation dairy cows enter positive energy balance sooner when supplemented with CLA, this experiment aimed at considering how this impacts the milk proteomes. For longitudinal comparisons, transition milk and mature milk were selected for this study as they provide unique comparable dataset.

## Results

A total of 479 unique proteins were identified in d 1 transition milk and mature milk samples based on having at least 2 unique peptide identifications per protein, 1% peptide false discovery rate, and presented in at least 50% of the samples (Supplementary S1). The identified proteins covered a wide range of protein classes involved in casein micelles, immunity, metabolic processes, and signaling, including immunoglobulin domains, lipoprotein constituents, chaperones, and proteases, located in various cell components, including membranes, lipoprotein particles, cytosols, vesicles, endoplasmic reticulum, lysosomes, and secreted proteins (Supplementary S2).

**Fig. 1:**
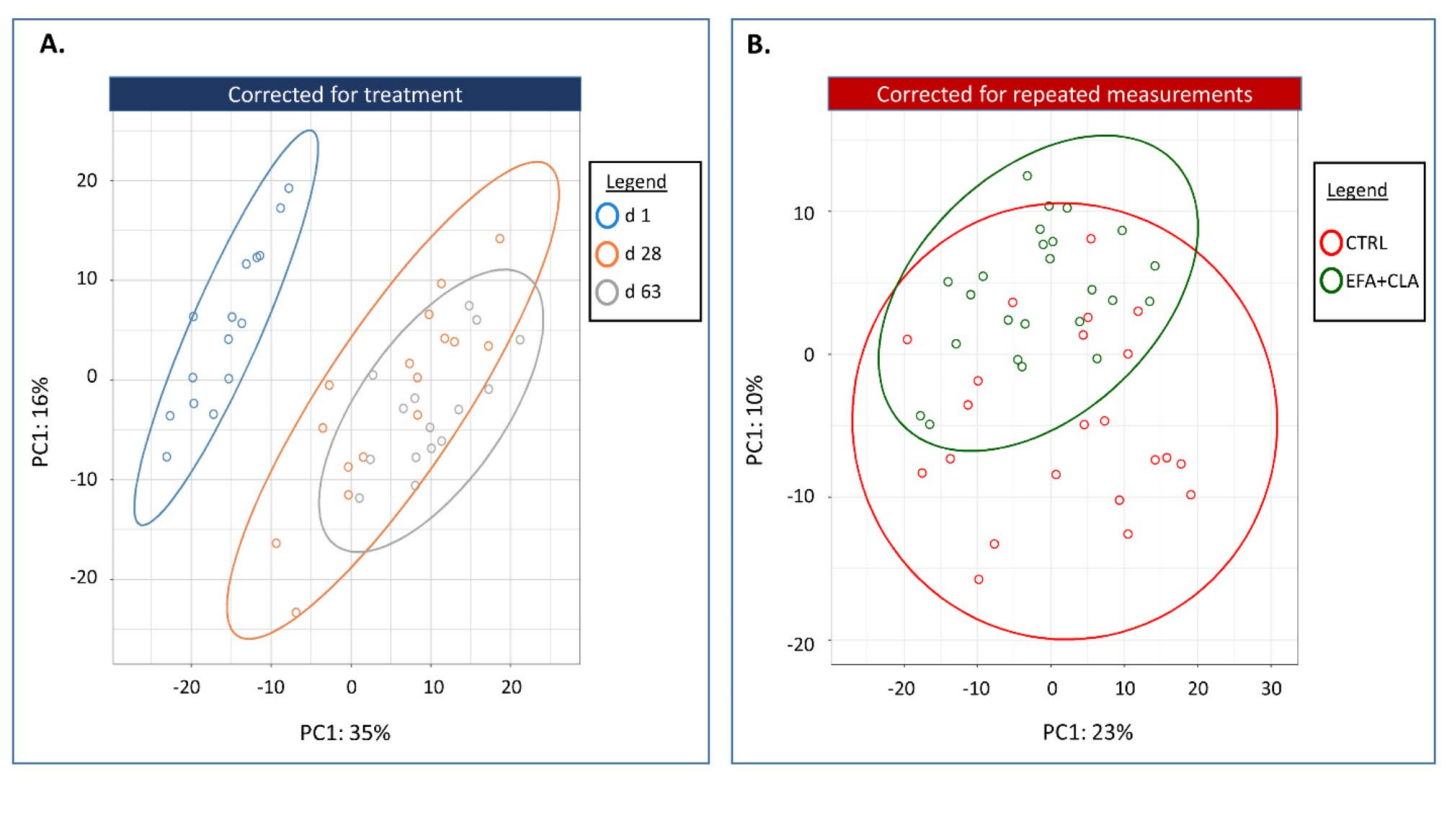
Principal component analysis (PCA) of the proteome in transition milk and mature milk from dairy cows receiving either no supplement or a combination of essential fatty acids (EFA) and conjugated linoleic acids (CLA). The first two principal components are plotted. (**A**) PCA was executed on the log2-transformed data within different time point. (**B**) PCA was executed on the log2-transformed data between CTRL and EFA + CLA groups. Colours indicate different timepoints/treatments. Percentages of variation explained by each PC are displayed along the axes.

Multilevel multivariate approaches were used to describe the complex structures of the repeated measure data, which clustered (unsupervised) and discriminated (supervised) sources of variation “lactation day” and “EFA + CLA supplementation”. Principal component analysis (PCA) outlined the primary pattern in milk protein abundance within time and between treatment groups. The PCA scatterplot of the milk proteome for the first and second principal components, which accounted for 50% of the total variation over time and 30% between treatments, is shown in Fig. 1. Based on the PCA analysis, the primary patterns within the dataset were between transition milk (d 1) and mature milk (d 28 and 64) regardless of the treatment effect. On the other hand, the impact of EFA + CLA was not strong and did not form any separable cluster (Fig. 1B).

### Proteome investigation throughout early lactation

**Fig. 2:**
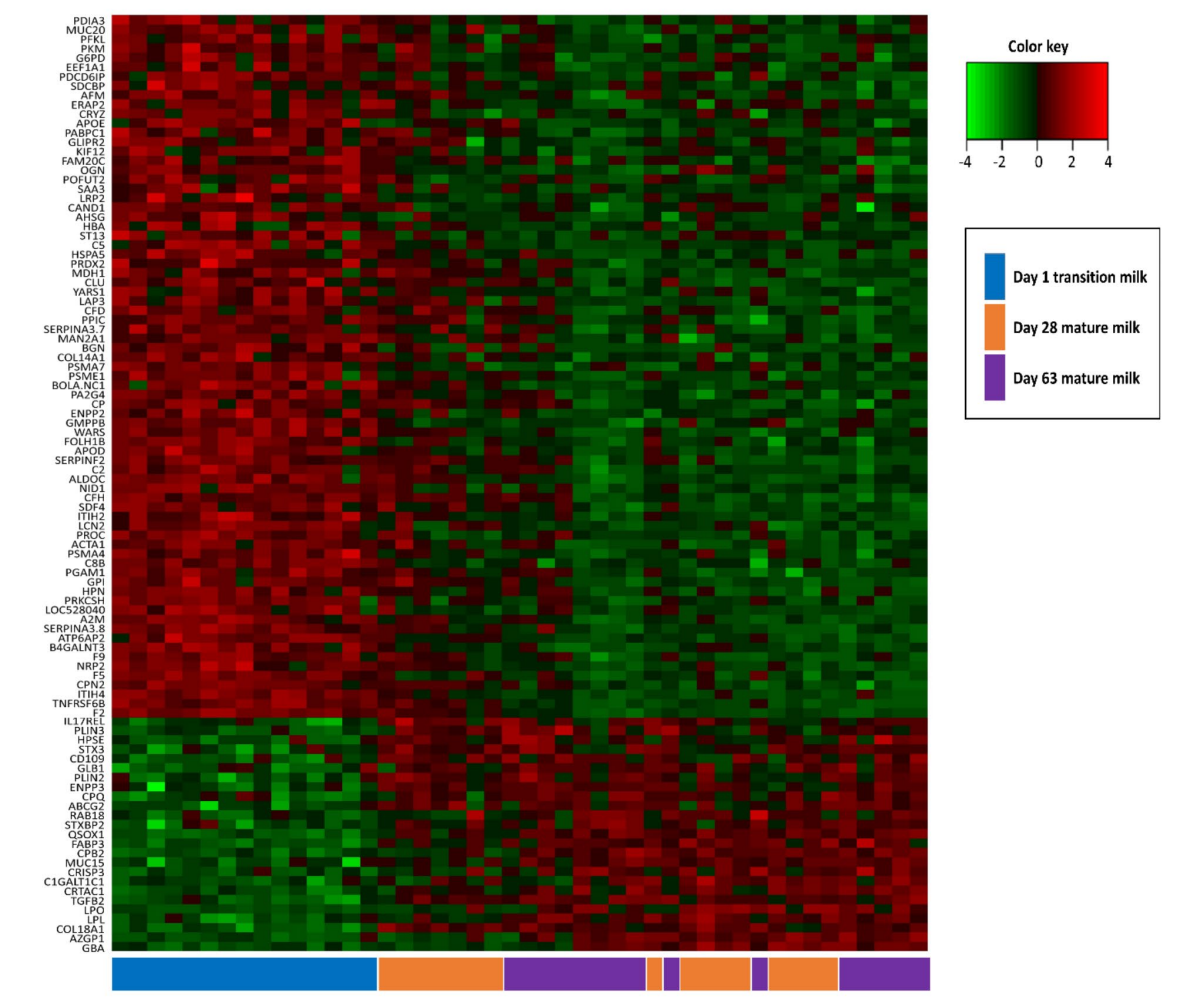
The top 100 discriminant proteins during various days of lactation (Time-DP) are shown in a heatmap. Red and green shadings represent high and low abundances, respectively. Time clustering with similar protein abundances is indicated by boxes below the sample numbers.

There were significant differences in the milk proteomes between transition and mature milk, whereas no differences were recorded in mature milk from d 28 and d 63 of lactation. Therefore, in the following chapter, the main focus will be comparing between d 1 transition milk and (d 28 and d 63) mature milk. Only the top 100 proteins ranked by partial least-squares discriminant analysis (PLS-DA) based on variable importance in projection (VIP) scores on component 1 were considered as time discriminating proteins (Time-DP) including 75 increased and 25 decreased proteins. Time-DP are shown by the heatmap in Fig. 2 (log2 transformed), and in Supplementary S3.

**Fig. 3:**
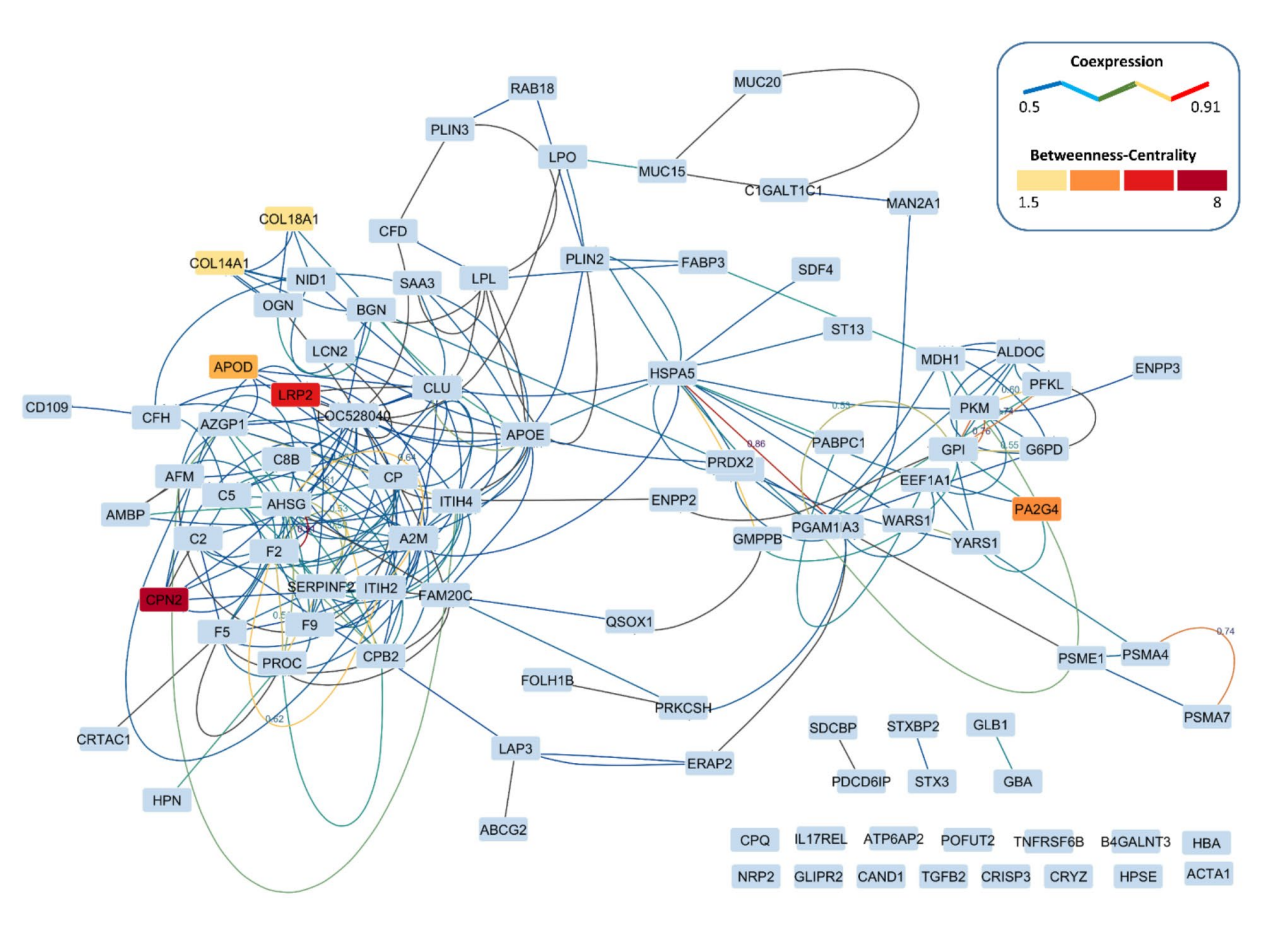
Protein-protein interaction network between the top 100 time discriminating proteins (Time-DP) between transition milk and mature milks (days 28 and 63). Time-DPs are highlighted by boxes with color density corresponding to their Betweenness-Centrality (which identifies important proteins playing a “bridge spanning” role in a network), while the edges between them are colored according to their co-expression (which identifies genes with similar expression patterns under physiological conditions).

Bioinformatics analyses including network analysis and gene ontology (GO) functional enrichment analysis were performed to gain a holistic view of the Time-DP interactions, and their biological process (BP), cellular component (CC), and molecular functions (MF). The Time-DP protein-protein interaction network is highlighted in Fig. 3.

The network analysis identified carboxypeptidase N subunit 2 (CPN2), low density lipoprotein receptor-related protein 2 (LRP2), apolipoprotein D (APOD), Alpha 2-HS Glycoprotein (AHSG), coagulation Factor II (F2), inter-alpha-trypsin inhibitor heavy chain H2 (ITIH2), alpha 2-antiplasmin (SERPINF2), and complement component 8 (C8B), as proteins that play a central role in network formation, and are probably crucial to control the communication among the transcriptional regulatory mechanisms.

Gene ontology and functional enrichment analysis of Time-DP revealed that protein-protein interaction (PPI) enrichment was significant (*P* < 1.0e-16). Based on molecular function, the majority of Time-DP have enzymatic peptidase activity (subclasses of endopeptidase, exopeptidase, metallopeptidase, carboxypeptidase, and hydrolase) or binding activity (Supplementary S4).

**Fig. 4:**
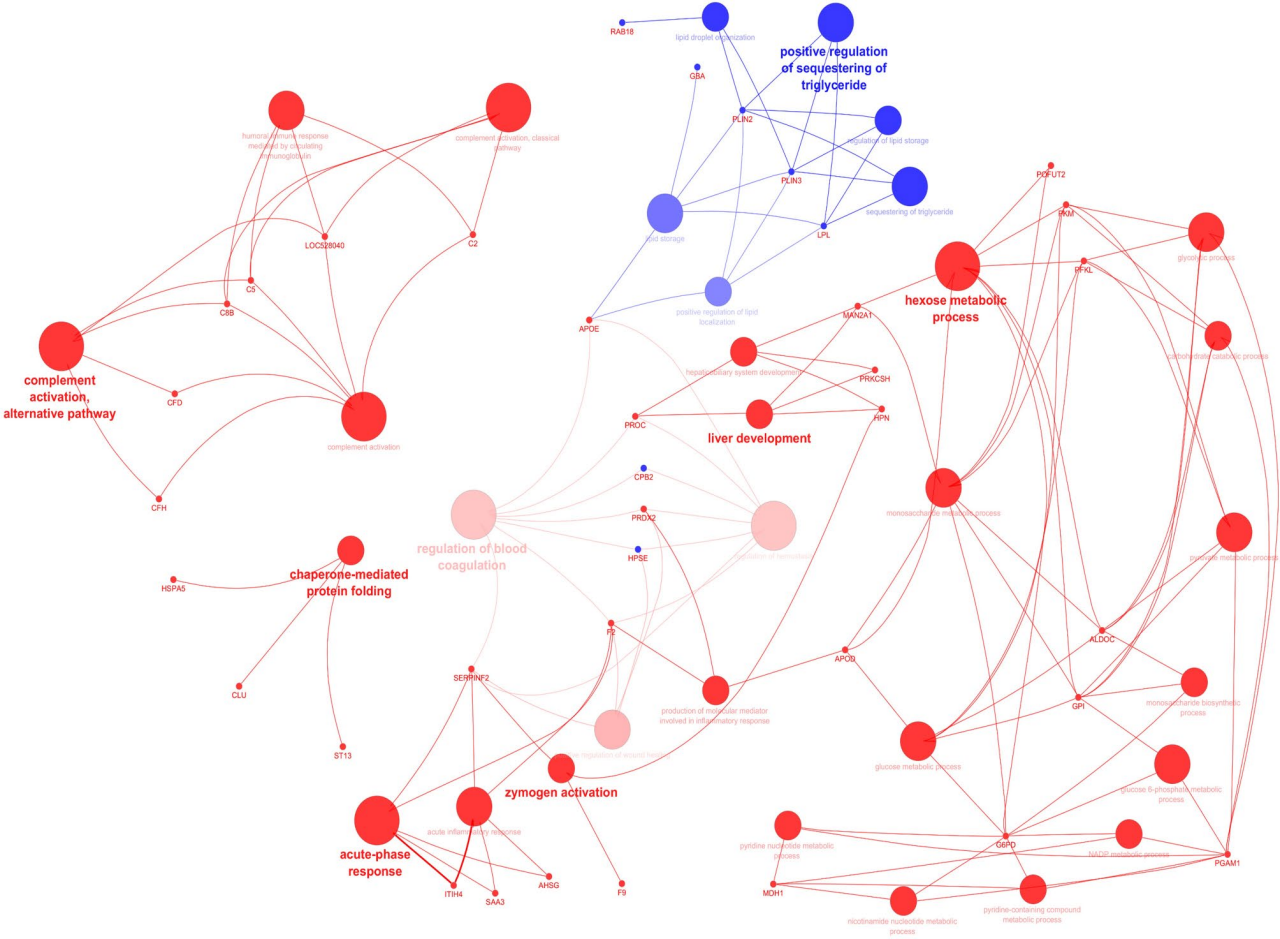
The network shows the biological process (BP) annotated by time discriminating proteins. Pathways are represented by different colors indicating annotated to upregulated (red) or annotated to downregulated (blue). In each pathway, the color shading (from light to dark color) represents the percentage of associated genes (low to high). Important genes that were initial for pathway enrichment or shared between pathways are highlighted (upregulated: red, downregulated: blue).

At the BP level, over-abundant proteins were annotated to the immune system process, chemokine production, acute-phase response, complement activation, carbohydrate derivative metabolic process, blood coagulation, hexose metabolic process, proteolysis, zymogen activation, and chaperon mediated protein folding (Fig. 4, Supplementary S5). On the other hand, under-abundant proteins were annotated by the regulation of lipid storage and their localization.

**Fig. 5:**
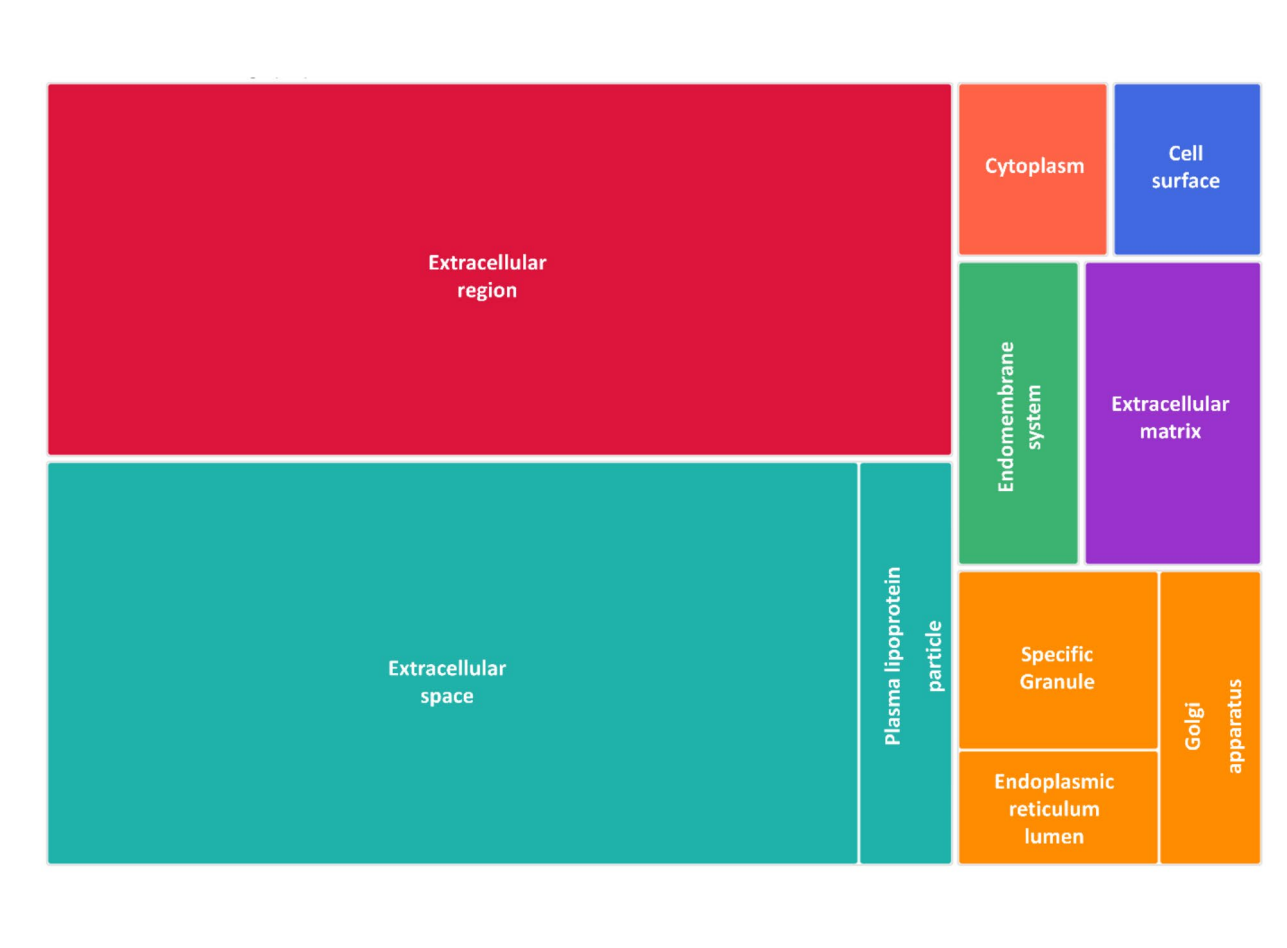
Time discriminant proteins are visualized as a treemap by indicating at which cellular component they are located. The size of each block corresponds to the number of identified proteins in that component. Colors indicate clustered components based on their similar localization.

At the cellular component level, Time-DP mainly consisted of proteins known to be secreted or present within the extracellular space but also present in the cell cytoplasm and Golgi (Fig. 5, Supplementary S6).

## Milk proteome affected by supplementation of fatty acids

**Fig. 6:**
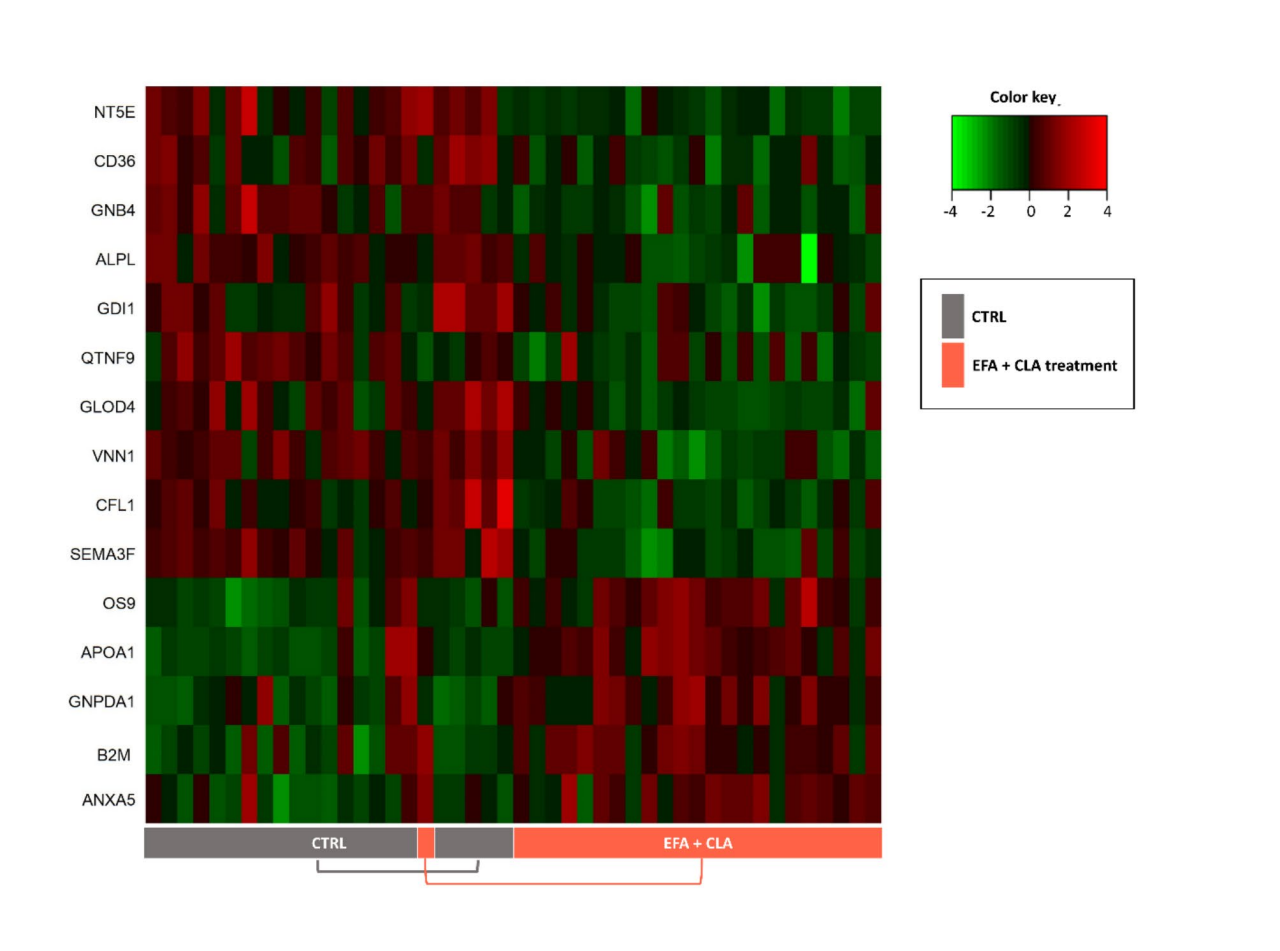
The top discriminating proteins (FA-DP) between cow milk supplemented or not with essential fatty acids (EFA) plus conjugated linoleic acids (CLA) are illustrated in a heatmap. Data were pooled over days within treatment. Red and green shadings represent high and low abundances, respectively. Treatments are indicated by boxes below the sample numbers.

As previously shown in Fig. 1, FA supplementation had a much smaller impact on the proteome profiles in transition and mature milks than the time effect. According to PLS-DA and VIP score, only 15 proteins were identified as FAs supplementation discriminating proteins (FA-DP), including 10 over-abundant and 5 under-abundant proteins (Supplementary S7). The heatmap in Fig. 6 shows the FA-DP.

There were no MF, CC, or BP annotations associated with FA-DP based on GO and functional enrichment analysis. However, according to UniProt databases, APOA1, CD36, 5′-nucleotidase (NT5E, also known as CD73), alkaline phosphatase ALPL, and pantetheinase (VNN1) are all associated with lipoprotein binding and assembly. Only APOA1 was more abundant among those in the EFA + CLA treated group.

## Discussion

There is growing evidence that supplementing dairy cows with ALA and CLA may alleviate postpartum NEB and metabolic pressure^11,18^. However, little is known at the molecular level on how this impact is reflected in the milk proteome profile over the first two months of lactation and what opportunities may exist to enrich milk to support neonatal development. This study investigated the transition of the proteome fingerprint in milk over the first nine weeks of lactation and its interaction with EFA + CLA treatment.

The composition and nutritional characteristics of the mammary secretions change drastically from colostrum to transition milk, then to mature milk within the first week of lactation^2,31^. Under the unique experimental design, we have previously reported the colostrum, transition milk, and mature milk characteristics during the first 8 weeks of lactation^14,19^. In brief, we observed that as the milking number increased, dry matter and protein content of colostrum and transition milk decreased. A similar pattern was observed in milk energy content. Also, we showed that milk protein and fat content plateaued after 3–4 weeks^14^. Furthermore, IgG1, IgG2, and IgM concentrations in colostrum were significantly higher and gradually decreased within a few days^32^, while lactose showed the reverse trend^19^.

Using skim samples the proteomes of colostrum and mature milk, as well as colostrum and transition milk were compared previously^8,9,33^. Complementary to those experiments, we compared transition milk (collected on d 1 postpartum) with mature milk collected on days 28 and 63. To expand the range of identified proteins, low abundance proteins were enriched before untargeted proteomics analysis. As a result, we identified 479 unique proteins, of which 150 proteins (31%) were also found in previously reported databases^8,27,33^, while the rest were identified only after enrichment.

The top 100 Time-DP were ranked, half of which serve as binding proteins (ion binding, proteoglycan binding), the other half as catalytic proteins. The same ontological groupings were observed when comparing milking numbers^8^. In milk fluids, most proteins are present in a bound form whether packaged in membrane-bound structures such as micelles, globulins, lipoproteins, and vesicles or bound with ions, proteins, and lipids to protect them from proteolysis and aggregation. In addition, the binding/packaging ensures protein structural stability and functional activity, and specifically fuses with the membranes of their target cells. A group of time-DP, including clusterin (CLU), heat shock (HS) protein 70 family protein 5 (HSPA5), and HS interacting protein (ST13), function as chaperones or chaperone-like proteins. Chaperones maintain protein homeostasis by recognizing unfolded, aggregated, or unstable polypeptides to protect other proteins against damage/aggregation^34^. HSPA5 regulates milk biosynthesis and proliferation of bovine primary mammary epithelial cells via mammalian target of rapamycin (mTOR) signaling^35^. The higher abundance of chaperones and binding proteins in milk from early milking might be structurally necessary to handle the higher concentration of nutrients and enzymes in transition milk than in mature milk.

On the other hand, colostrum and transition milk contain more minerals, including Ca, Mg, P, Cu, Se, Zn, Fe, Na, and K as compared to mature milk^36^. Free-form ion minerals may induce toxic reactions, protein aggregation, and coagulation^37^. Therefore, most minerals are usually found in body fluids in conjunction with specific binding proteins or protein cofactors, such as molecular chaperones or transporters^38^. Additionally, the high expression of binding proteins may contribute to the higher concentration of specific ions in transition milk to prevent the free, ionized state in the fluid.

The innate and adaptive immunity of newborn calves is not fully developed and gradually matures over time, making them immunosuppressed and vulnerable. This is why they depend heavily on colostrum and milk to develop their immune systems^39^. Previously, the proteins critical to the calf’s innate immune system were highlighted comprising complement factors, collectin, and acute phase protein (APP) families^7^. Accordingly, our GO analysis revealed several Time-DPs involved in defense or immunity, particularly acute phase response, inflammatory response, antioxidant activity, complement activation, and humoral immune response mediated by circulating immunoglobulins. Immune-related proteins such as AHSG, ITIH4, SAA3, SERPINF2, APOD, F2, CFH, CFD, C2, and C5 declined over time, in line with previous reports^8,33^. A previous study suggested that these low-abundance immunomodulators are important for developing the calf’s immunity^7^. In addition, these immune modulatory proteins also seem crucial to gut development and establishing the gut microbiota in calves^39^, however, their abundances decreases when the calves’ gut closure occurs within a day after birth^40^. The specific impact of these immune modulatory proteins/families on the immune system of neonatal calves needs to be further investigated in future studies. In addition, colostrum and transition milk provide proteins that support neonatal gastrointestinal maturation^33,41^. In this regard, some of the Time-DP were growth factors or signaling molecules probably involved in ongoing gut development. For instance, the relative abundances of transforming growth factor (TGF)-β2 and membrane-bound mucins (MUC15 and MUC20) were higher in transition milk than in mature milk.

Several Time-DPs have enzymatic functions, such as peroxidases and lipases. Among them, lactoperoxidase (LPO), as a member of the mammalian heme peroxidases that catalyze a wide range of intermediate compounds with antimicrobial activity^42^, had a lower abundance in day 1 transition milk (compared to mature milk). The same trend was observed for lipoprotein lipase (LPL), which is responsible for the hydrolysis of TG into free FAs^43^. Previously, these two enzymes were shown to be less abundant in initial milk secretions, to increase rapidly until reaching a plateau, and then remaining constant in milk during early lactation^44^.

LPL was also among another cluster of DP related to TG and lipoprotein assembly, TG lipolysis, lipid droplet organization and localization, and cholesterol metabolism. Milk fats, consisting largely of TG, are found in MFG, an emulsified globular structure surrounded by a delicate membrane called MFG membrane (MFGM) to protect them from lipases^45^. The MFG is a complex structure containing a mixture of unique proteins to control its size and ensure its stability^17^. Previously, it has been shown that the size distribution of bovine MFG and the FA profile of milk changes by milking number^46^ also in association with LPL activity^47^. LPL is mainly found in skim milk but also attached to the MFGM^47,48^. Several factors such as lactation stage (early and late)^43,49^, diet^50^, and hormones^51^ influenced the LPL activity and transcript abundance in mammary glands and adipose tissues. In this regard, LPL expression is shown to be selectively induced by transcription factor, peroxisome proliferator-activated receptor gamma (PPARγ), which regulates systemic lipid and glucose metabolism^52^. PPARγ is highly abundant in bovine mammary epithelial cells and its expression is induced during lactation^53^, suggesting involvement in milk fat synthesis^54^. Therefore, it is possible that LPL expression is also under the regulatory mechanisms that control milk fat synthesis. Despite not isolating MFG for this study, some of the identified time-DP were previously reported to be part of the MFG constituents such as mucins (MUC15, MUC20), and fatty acid binding proteins (FABP3)^55^. Freezing and thawing or acid treatment probably caused spontaneous lipolysis in cow’s milk, which subsequently damaged the structure, integrity, and stability of MFGM^47,50^. Accordingly, it is possible that MFG proteins are denatured and bound to whey protein, which is why they can still be detected in skim milk.

A difference in the fat content between colostrum and milk likely affects the assembly and size of milk droplets, in which identified proteins are likely to play a vital role. For instance, it has been reported previously that FABP3 negatively correlates with the size of MFG^17^. Also, the content of perilipins in milk is proportional to its lipid content and may play an important role in the synthesis and secretion of milk lipids^56^. We observed a lower abundance of perilipins (PLIN2 & PLIN3) in day 1 transition milk compared to mature milk.

As lactation progresses, the milk proteome along with milk fat, protein, and ion content changes. Part of the time-DPs were related to the immune system and gastro-intestinal tract development and regulating fat and energy metabolism, all of which require modified structural and binding proteins in order to alter the biophysical characteristics of milk globules. Alterations in the milk proteome may be aligned to mechanisms that control milk composition to meet calves’ needs during different stages, which require further investigation.

FA supplementation, especially CLA, may also interfere with milk composition during lactation. We have previously reported that CLA (in combination with EFA) supplementation induced milk fat depression, altered milk FA composition, and reduced energy-corrected milk in early lactation^14^. However, milk yield, protein, and lactose were unaffected by FA treatment. Also, elevated concentrations of ALA and CLA in cow plasma and milk were positively and strongly correlated with their concentration in calves’ plasma^19^. Maternal EFA + CLA supplementation had no effect on calves’ plasma metabolite and hormone concentrations including glucose, β-hydroxybutyrate (BHB), total protein, urea, triglyceride, non-esterified fatty acids (NEFA), insulin, cortisol, growth hormone (GH), insulin-like growth factor (IGF)-I, IGFBP-2, and IGFBP-4^57^. Moreover, maternal supplementation of CLA caused no differences in plasma inflammation markers consisting of total protein, albumin, interleukin (IL)-1β, IL-6, and haptoglobin^58–60^.

Supplementation with EFA and CLA affected only a small number of proteins in skimmed milk, involved in the molecular regulation of lipoprotein particles and MFG assembly. A number of the FA-DPs have previously been reported to be affected in skim milk^27^ and MFGM^17^ of CLA-supplemented cows including CD36, APOA1, GLOD4, and NT5E. It has been reported previously that the machinery of lipoprotein assembly is highly sensitive to dietary modifications and coordinates protein synthesis based on lipid substrate availability^61^. Also, there is a negative correlation between PUFA content (FA saturation) and MFG size^46^. In this regard, a recent study found that supplementing CLA at mid-lactation decreased MFG size in dairy cows^15^. Among FA-DP, CD36 and ANAX5 have previously been identified in dairy cow MFG^62^, suggesting they may participate in the pathways related to the formation and integrity of MFG. In particular, CD36 is a scavenger receptor which plays an important role in high-affinity tissue uptake of long-chain FAs and lipoproteins into mammary epithelial cells, where they can be used for *de novo* FA synthesis or packaging into MFG^63^. In addition, a correlation was also found between the protein abundance of APOA1 and GLOD4 and the MFG size parameters of dairy cows^17^. APOA1 serves as a major component of the apolipoprotein structure, especially regulating the high density lipoprotein (HDL) particle size^64^, responsible for the proper incorporation and stabilization of FA in (MFG) membranes. Therefore, enriching supplemented FA in milk (such as in MFG) may require a modified lipoprotein assembly and a lipid binding and transport system, which consequently affects MFG size and distribution.

We recently discussed that higher concentrations of EFA and CLA may impact both tissue lipogenesis and lipolysis through ligand activation of transcriptional regulatory pathways such as sterol response element binding proteins (SREBPs) and PPAR^11^. CLA reduces milk fat synthesis by influencing PPARγ signaling and consequently genes involved in mammary lipogenesis^65,66^. In this regard, several FA-DP, including APOA1, CD36, and VNN1, were identified under the PPAR signaling pathway^67,68^, which might suggest the contribution of more complex energy and lipid metabolism regulatory networks. However, further research is needed to fully understand the underlying feedback mechanisms in the mammary gland, liver, adipose tissue, and their interaction.

## Conclusion

This study provided a comprehensive understanding of the proteome alterations in dairy cows’ skim milk during the first nine weeks of lactation and how it is affected by FA, in particular, ALA and CLA supplementation. In the first weeks of lactation, the milk proteome signature is affected in line with changes in milk protein, fat, and ion concentrations. After reaching peak production, the protein profile remained unchanged regardless of variations in energy balance. Compared to mature milk, transition milk contains specific proteins such as immune regulatory proteins and growth factors that are likely to be involved in the calves’ immune system and gut development. The differential abundance of binding proteins and chaperones in transition milk may contribute biophysically and structurally to the reassembly of milk condensed constituents while protecting enzymes from unwanted actions/aggregations. Supplementation with CLA and EFA had only a minor impact on constituents of lipoprotein assembly that are probably necessary for the synthesis, transfer, and rearrangement of the supplemented FA into milk lipid components. Collectively, the skim milk proteome seems dynamically affected by the cow’s metabolic physiology at the onset of lactation but also by enriching specific FA, which might open up new opportunities for future calf preweaning nutrition.

## Materials and methods

### Animals, treatments, and sampling

For all experimental procedures, animal welfare guidelines were followed and approved by the relevant authority, i.e., the Department for Animal Welfare Affairs of the State of Mecklenburg-West Pomerania, Germany (Landesamt für Landwirtschaft, Lebensmittelsicherheit und Fischerei; LALLF M-V/TSD/7221.3–1–052/15). The experimental design has been previously described elsewhere^14^. In brief, 16 Holstein dairy cows in their second lactation were randomly assigned to one of two treatments that started from 63 days before and lasted until 63 days after parturition. The control treatment (CTRL, *n* = 8) was coconut oil (76 g/d) free from essential fatty acids (EFA) and conjugated linoleic acid (CLA). The second treatment (*n* = 8) was a combination of linseed oil (78 g/d), safflower oil (4 g/d), and Lutalin^®^ (CLA, cis-9, trans-11: 10 g/d; trans-10, cis-12: 38 g/d), formulated to compensate for the energy intake of the CTRL treatment, while providing EFA and CLA (EFA + CLA). During the dry period, each dose was halved. Treatments were provided as two equal doses of oil supplements infused into the abomasum twice daily at 0700 and 1630 h. The cows were housed in free-stall barns with ad libitum access to a corn silage-based total mixed ration (TMR), formulated according to recommendations provided by the Society for Nutrition Physiology^69–71^ and Deutsche Landwirtschaftliche Gesellschaft (DLG, 2013), for antepartum and postpartum^72^.

The cows were milked twice daily and the first milking after parturition was considered as colostrum. Milk from the second milking up to the end of d 5 after parturition was defined as transition milk. Milk yield was recorded electronically after each milking. The composition of colostrum, transition milk, and milk comprising protein, fat, and lactose was reported previously^19^. The chemical composition of colostrum and in transition milk was determined using classical chemical methods^19^. For mature milk an infrared spectrophotometric method (MilkoScan FT6000, Foss GmbH, Hamburg, Germany) was used. The statistical comparisons of milk and energy variables are summarized in Supplementary S8. Ingredients, the chemical composition of the experimental diets, and cow milk parameters including FA composition are reported previously^14,19^.

From each cow, individual samples of colostrum, transition milk, and milk (5 mL) were taken and stored at -70 °C until further chemical analysis. The proteomics analysis used transition milk (only second and third milking) and mature milk (from days 28 and 64). As colostrum protein preparation demands ultracentrifugation, we chose the second and third milkings, i.e., transition milk that is close to colostrum (based on our previous measurements^19^), but can be prepared in a comparable manner as mature milk.

## Milk protein extraction and enrichment

The processes for milk protein extraction and enrichment, and peptide preparation were described previously^50^. Briefly, frozen raw milk samples were slowly defrosted overnight at 4 °C, then they were homogenized with incubation at 37 °C for 1 h. For defattening, samples were centrifuged at 3500 *g* for 15 min at 4 °C and the fat layer on top was removed carefully using a spatula. Adding acetic acid (1 N) precipitated casein from skim milk at pH ∼ 4.6. After centrifugation at 10 000 *g* for 10 min at 4 °C, the supernatant was recovered. The presence of high Igs and caseins content in transition and mature milks may prevent the screening of low abundance proteins, which might provide high information with differential abundance. A ProteoMiner Small-Capacity column (#163–3006, Bio-Rad Laboratories, Hercule (CA)) was used to enrich skim milk proteins with minor or low abundance according to the manufacturer’s instructions. The ProteoMiner technology enables the enrichment and detection of mid-to low abundance proteins by reducing the dynamic range between proteins with various abundance, resulting in a wide range of low to high abundance proteins being identified^73^. The application of ProteoMiner increased the number of identified proteins in a preliminary experiment by 160% (Supplementary S9). The bicinchoninic acid (BCA) assay kit (Thermo Scientific, Rockford, IL, USA) measured skim milk protein concentration using bovine serum albumin as a reference protein standard.

## Protein digestion and peptide analysis on nano-LC-MS/MS

Enriched proteins were reduced, using sodium dodecylsulfate buffer (4%) with dithiothreitol (20 mM), alkylated, using iodoacetamide (50 mM), and concentrated using S-Trap^®^ column (Protifi, Fairport, NY) according to the manufacturer’s instructions. Proteins trapped in S-Trap columns were digested by adding 2 µg of sequencing grade trypsin (Promega, Madison (WI)) at a ratio of 1:50 [w/w] enzyme/protein in triethylammonium bicarbonate (50 mM) to the filter for overnight at 37 °C, for details see^74^. The resulting peptides were eluted in two steps after digestion: first, by adding 40 µL of 50 mM TEAB, 0.2% formic acid in H_2_O; second, by adding 50 mL of 50% acetonitrile, 0.2% formic acid in H_2_O. Finally, peptides were dried in a Speed Vac (Eppendorf AG, Hamburg, Germany) for 1 h and suspended in 20 µL of equilibration solution (H_2_O /trifluoroacetic acid − 99.95/0.05).

The milk peptides were analyzed by label-free LC-MS/MS quantitative proteomics approach using an Ultimate 3000 RSLC system (Thermo Fisher Scientific) coupled to a Q Exactive HF-X mass spectrometer (Thermo Fisher Scientific), according to the previously described method by^75^. In brief, 1 µL of the equilibrated peptides was injected onto analytical column (Acclaim PepMap 100 –75 μm inner diameter × 25 cm length; C18–3 μm -100Å - SN 10711311) operating at 400 nL/min for separation of peptides. Peptides were eluted from analytical column to nanoelectrospray ion source (Proxeon) using the following gradient: 96% A solvent and 4% B solvent for 6 min, followed by a gradual increase of the B solvent to 20% for 70 min. To perform MS/MS analysis, the following set-up was used on the Orbitrap system: the nanoelectrospray ionization source was set to positive ion mode at 1.6 kV; MS1 with a resolution of 60,000 and an injection time of 50 ms in the range of 375 to 1600 m/z; 18 MS2 scans at 15,000 nm and 100 nm injection time.

### Data acquisition, processing, and statistical analysis

Using Progenesis QI software (version 4.2, Nonlinear Dynamics, New Castel upon Tyne, UK), MS/MS spectrum was first aligned to the reference sample, assigned automatically by the program as having the highest coverage of peptide ions, and then processed for peptide ions identification using default parameters (ion charged to five and Ions ANOVA P-value < 0.05). MASCOT (version 2.5.1) interrogation engine was used to identify corresponding proteins according to the following setup: *Bos taurus* decoy database (Uniprot, download date: 2020, a total of 37,513 entries), trypsin as a digest enzyme, tryptic specificity to cleavage C-terminal after lysine or arginine residues, allowing two missed cleavages, variable modification to carbamidomethylation (C) and oxidation (M), mass tolerance to 10 ppm for precursor ions and 0.02 Da for fragment ions, false discovery rate (FDR) < 0.01, over two unique validated peptides.

The protein intensities were log-transformed, and missing intensities with a frequency less than 50% of samples were removed. After a quality control procedure, two individual samples (d 1 and 63) were outliers with high leverage and were excluded from the analysis. In order to deal with repeated measurements, the sources of variation were modelled separately over time and between treatments using a multilevel multivariate analysis that includes correction for PCA and PLS-DA. A comparison of this procedure with a regular multivariate approach has shown that it significantly increases the accuracy of feature selection and/or classification^76,77^. To control PLS-DA overfitting, only the top discriminating proteins based on variable importance in projection (VIP) scores on component 1 of PLS-DA were selected and were checked to meet the FDR < 0.05. Statistical analysis was performed in mixOmics R-package and graphed using ggplot2 R-package in R statistical software (R version 4.0.0). The most discriminating proteins are listed in Supplementary S1.

The GO categorization, including BP, MF, and CC were conducted under *Bos taurus* databse using ClueGo (v 2.5.10) in Cytoscape (v 3.10.0). The important proteins within each pathway are highlighted as being shared among some pathways or as initial to form clusters using CluePedia (v 1.5.10). Network analysis was performed using STRING (v 2.0.1) app and Analysis Network tool in Cytoscape set for undirected networks (using default parameters). The betweenness centrality index is determining the most relevant nodes that provide comprehensive centrality information about proteins and how they communicate (communication flow). Accordingly, proteins (nodes) with a higher betweenness centrality score are bioinformatically expected to maintain functionality and coherence of signalling mechanisms. The co-expression values, on the other hand, represents the degree of similarity between expression profiles of all genes (proteins) in a particular set of biological samples. A co-expression network allows the identification of functional interactions between genes that are under common regulatory control^78^.

## Electronic supplementary material

Below is the link to the electronic supplementary material.

Supplementary Material 1

Supplementary Material 2

Supplementary Material 3

Supplementary Material 4

Supplementary Material 5

Supplementary Material 6

Supplementary Material 7

Supplementary Material 8

Supplementary Material 9

## Data Availability

All the proteomics data and the related analyses are available as supplementary files.
