# Supplementary material for: The skimmed milk proteome of dairy cows is affected by the stage of lactation and by supplementation with polyunsaturated fatty acids
Source: Sci Rep. 2024 Oct 14;14:23990. doi: 10.1038/s41598-024-74978-1 (PMC11473731; doi:10.1038/s41598-024-74978-1)
Supplement: Supplementary file 8 — Supplementary Material 8 [file 41598_2024_74978_MOESM8_ESM.docx]

Supplementary table S2. Milk variables and markers of energy in dairy cows abomasaly supplemented with coconut oil (CTRL) or a mixture of essential fatty acids and conjugated linoleic acids (EFA+CLA) during antepartum and postpartum.

| Variable | week | Treatment | |  | P-value | | |
| --- | --- | --- | --- | --- | --- | --- | --- |
|  |  | CTRL | EFA+CLA |  | time | treatment | time * treatment |
| Milk Fat  (%) | 1 | 5.42 ± 0.97^a^ | 3.62 ± 0.81^b^ |  | <0.01 | <0.01 | 0.59 |
|  | 4 | 4.20 ± 0.64^a^ | 1.96 ± 0.45^b^ |  |  |  |  |
|  | 8 | 3.96 ± 1.10^a^ | 1.64 ± 0.41^b^ |  |  |  |  |
|  |  |  |  |  |  |  |  |
| Milk Protein  (%) | 1 | 3.87 ± 0.22 | 3.76 ± 0.25 |  | <0.01 | <0.01 | 0.14 |
|  | 4 | 3.11 ± 0.40 | 3.00 ± 0.23 |  |  |  |  |
|  | 8 | 3.12 ± 0.36 | 2.96 ± 0.23 |  |  |  |  |
|  |  |  |  |  |  |  |  |
| Milk Fat/Protein | 1 | 1.40 ± 0.25^a^ | 0.96 ± 0.20^b^ |  | 0.02 | <0.01 | 0.22 |
|  | 4 | 1.37 ± 0.26^a^ | 0.66 ± 0.17^b^ |  |  |  |  |
|  | 8 | 1.29 ± 0.40^a^ | 0.56 ± 0.17^b^ |  |  |  |  |
|  |  |  |  |  |  |  |  |
| Milk Lactose   (%) | 1 | 4.52 ± 0.20 | 4.64 ± 0.23 |  | <0.01 | 0.09 | 0.7 |
|  | 4 | 4.82 ± 0.06 | 4.85 ± 0.18 |  |  |  |  |
|  | 8 | 4.76 ± 0.07 | 4.83 ± 0.08 |  |  |  |  |
|  |  |  |  |  |  |  |  |
| Milk Urea  (mg/L) | 1 | 211 ± 34.1 | 126 ± 89.4 |  | 0.07 | <0.01 | 0.61 |
|  | 4 | 140 ± 40.8 | 69.8 ± 31.0 |  |  |  |  |
|  | 8 | 151 ± 42.1 | 102 ± 33.3 |  |  |  |  |
|  |  |  |  |  |  |  |  |
| Milk yield (kg) | 1 | 27.0 ± 3.24 | 26.82 ± 4.88 |  | <0.01 | 0.26 | 0.67 |
|  | 4 | 37.28 ± 6.74 | 40.41 ± 4.83 |  |  |  |  |
|  | 8 | 38.87 ± 8.25 | 41.41 ± 3.55 |  |  |  |  |
|  |  |  |  |  |  |  |  |
| Energy corrected milk (ECM, kg/d) | 1 | 32.52 ± 4.77 | 26.21 ± 5.08 |  | 0.07 | <0.01 | 0.76 |
|  | 4 | 37.26 ± 6.63^a^ | 29.45 ± 2.82^b^ |  |  |  |  |
|  | 8 | 37.66 ± 7.32^a^ | 28.59 ± 3.55^b^ |  |  |  |  |
|  |  |  |  |  |  |  |  |
| Energy balance (EB, MJ NE_L_/d) | 1 | -49.4 ± 18.2^a^ | -32.4 ± 13.8^b^ |  | <0.01 | <0.01 | 0.61 |
|  | 4 | -38.7 ± 14.4^a^ | -11.1 ± 9.41^b^ |  |  |  |  |
|  | 8 | -18.1 ± 21.3^a^ | 5.15 ± 12.2^b^ |  |  |  |  |
|  |  |  |  |  |  |  |  |
| FE/ECM^3^ | 1 | 2.93 ± 0.86 | 3.63 ± 3.56 |  | 0.03 | 0.85 | 0.47 |
|  | 4 | 2.39 ± 0.24^a^ | 1.85 ± 0.20^b^ |  |  |  |  |
|  | 8 | 2.02 ± 0.33^a^ | 1.6 ± 0.20^b^ |  |  |  |  |
|  |  |  |  |  |  |  |  |
| Energy efficiency | 1 | 1.22 ± 0.31 | 1.13 ± 0.41 |  | <0.01 | 0.01 | 0.65 |
|  | 4 | 1.04 ± 0.11^a^ | 0.80 ± 0.08^b^ |  |  |  |  |
|  | 8 | 0.88 ± 0.14^a^ | 0.69 ± 0.09^b^ |  |  |  |  |

Data are presented as least square means (LSM) ± standard deviation (SD); LSM with different lowercase letters (a, b) differ (P < 0.05) at the respective time point. The data analysis was conducted using R (v.4.0.0).
